# Supplementary material for: Androgen deprivation upregulates SPINK1 expression and potentiates cellular plasticity in prostate cancer
Source: Nat Commun. 2020 Jan 20;11:384. doi: 10.1038/s41467-019-14184-0 (PMC6971084; doi:10.1038/s41467-019-14184-0)
Supplement: Supplementary file 1 — Supplementary Information [file 41467_2019_14184_MOESM1_ESM.pdf]

Supplementary Information

Androgen deprivation upregulates SPINK1 expression and potentiates cellular plasticity in prostate cancer

Ritika Tiwari[1,†], Nishat Manzar[1,†], Vipul Bhatia[1], Anjali Yadav[1], Mushtaq A. Nengroo[2], Dipak Datta[2], Shannon Carskadon[3], Nilesh Gupta[4], Michael Sigouros[5], Francesca Khani[6], Matti Poutanen[7], Amina Zoubeidi[8], Himisha Beltran[9], Nallasivam Palanisamy[3], Bushra Ateeq[1*]

[1]Molecular Oncology Laboratory, Department of Biological Sciences and Bioengineering, Indian Institute of Technology Kanpur, Kanpur, 208016, U.P., India;
[2]Division of Cancer Biology, CSIR-Central Drug Research Institute, Lucknow, 226031, U.P., India;
[3]Vattikuti Urology Institute, Department of Urology, Henry Ford Health System, Detroit, MI 48202, USA;
[4]Department of Pathology, Henry Ford Health System, Detroit, MI 48202, USA;
[5]Division of Medical Oncology, Weill Cornell Medicine, New York, NY 10065, USA;
[6]Department of Pathology and Laboratory Medicine, Weill Cornell Medicine, New York, NY, 10065, USA;
[7]Institute of Biomedicine, Research Centre for Integrative Physiology and Pharmacology, University of Turku, Turku, Finland;
[8]Vancouver Prostate Centre and Department of Urologic Sciences, University of British Columbia, Vancouver BC, Canada;
[9]Department of Medical Oncology, Dana Farber Cancer Institute, Harvard Medical School, Boston, MA 02215, USA

[†]These authors contributed equally to this work.

Keywords: Prostate Cancer; ADT; SPINK1; AR; REST; SOX2; NEPC.

Financial support: This work is supported by the Wellcome Trust/ DBT India Alliance grant (IA/I(S)/12/2/500635 to BA).

***Corresponding Author**:
Bushra Ateeq, Ph.D.
Molecular Oncology Laboratory,
Department of Biological Sciences and Bioengineering,
Indian Institute of Technology Kanpur,
Kanpur, 208016, U.P., INDIA
Phone: +91 512 2594083
Fax: +91 512 2594010
Email: bushra@iitk.ac.in

Supplementary Figure 1

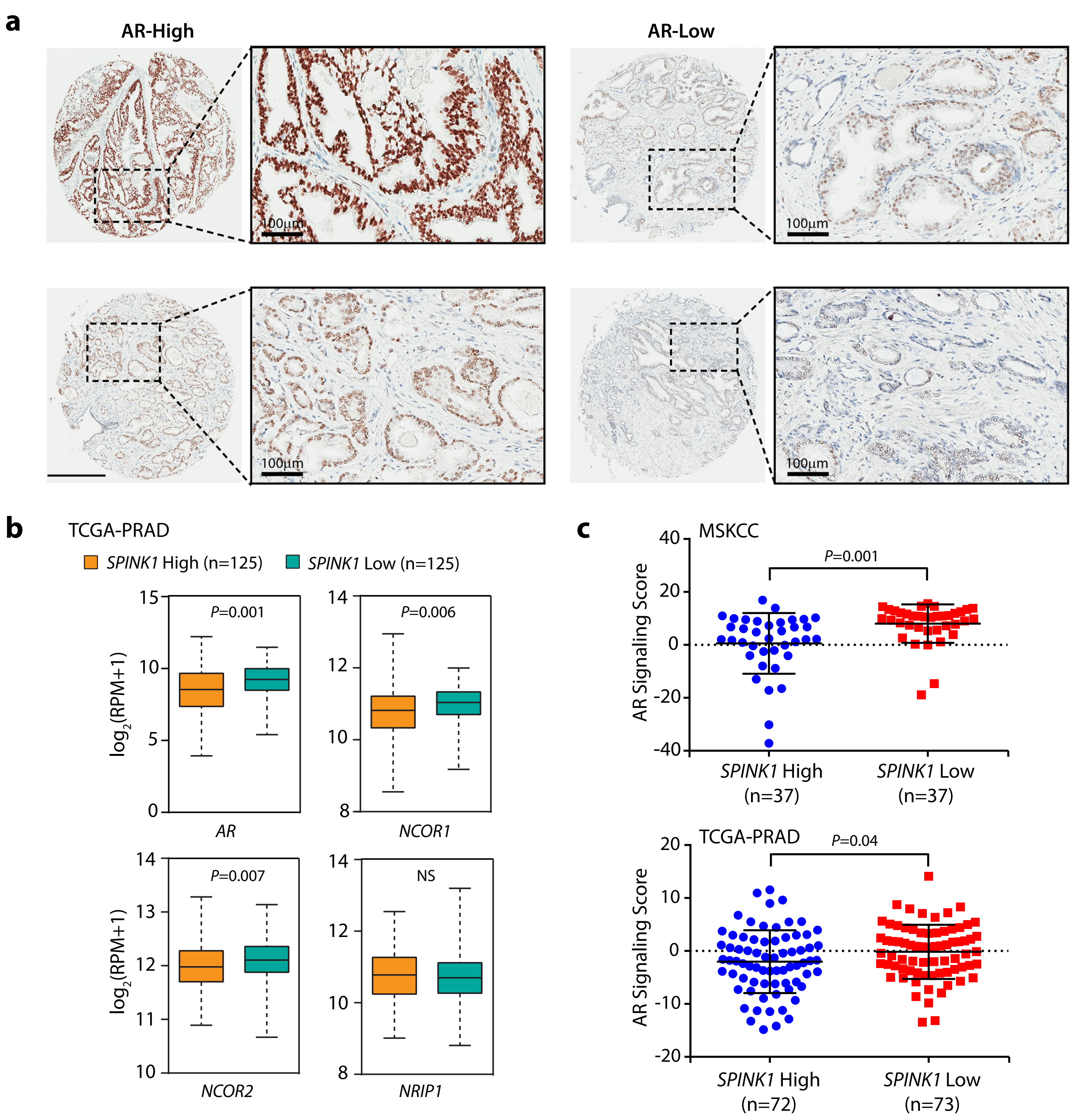

Supplementary Figure 1. *SPINK1* expression is negatively correlated with *AR* and members of AR repressive complex in PCa patients.
(a) Representative micrographs showing immunohistochemical (IHC) staining for AR in PCa tissue microarray (TMA) cores (n=237) wherein based on intensity, AR staining is categorized as high (Gleason score 8), medium (Gleason score 7), low (Gleason score 7) and negative (Gleason score 7). Scale bar represents 500 µm and 100 µm for the entire core and the inset, respectively. **(b)** Relative expression of the members of AR repressive complex (*AR, NCOR1, NCOR2* and *NRIP1*) in *SPINK1* high (n=125) and low (n=125) PCa patients, stratified by employing quartile-based normalization of *SPINK1* expression in TCGA-PRAD dataset. Transcripts level shown as $\log_2$ (RPM+1). **(c)** Dot plot depicting inverse association between AR signaling score and *SPINK1* expression in the MSKCC (n=74; top) and TCGA-PRAD datasets (n=145; bottom) in *SPINK1* high and low PCa patients, stratified by employing quartile-based normalization of *SPINK1* expression. AR signaling score represents the mean expression of 10-gene signatures linked to AR signaling.
Data for panel (**b**) is presented as box-and-whisker plots with median, where the box extends from 25th-75th percentile, and whiskers ranges from minimum and maximum values. For panel (**c**) data is presented as scatter plots with mean ± SD. Statistical significance for panels (**b**, **c**) was calculated using two-tailed unpaired Student's *t*-test.

Supplementary Figure 2

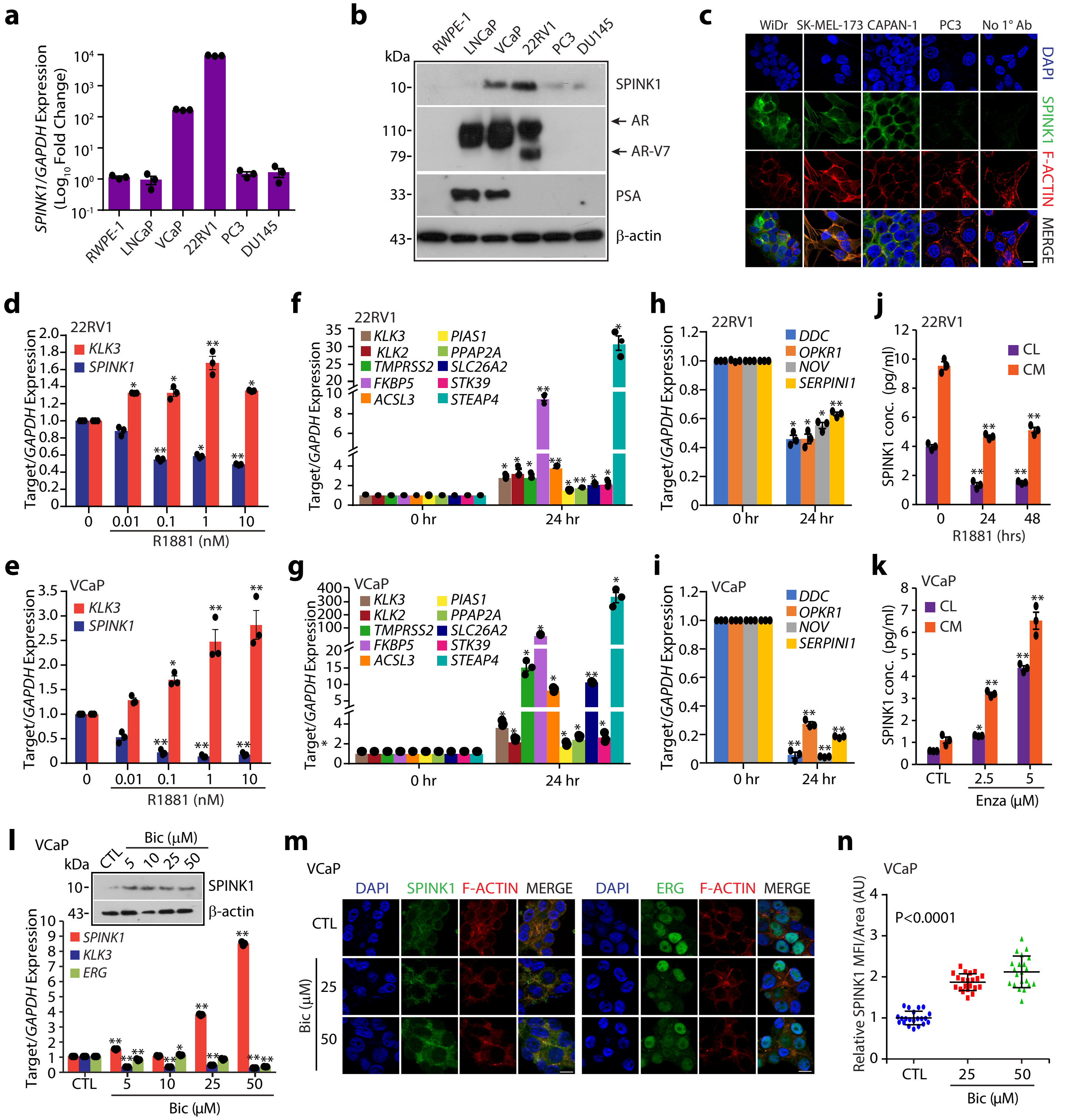

Supplementary Figure 2. SPINK1 expression is negatively regulated by androgen signaling in PCa cells.
(a) QPCR data showing relative expression of *SPINK1* in the PCa cell lines panel. **(b)** Same as in (a), except immunoblot analysis showing SPINK1, AR and PSA expression. β-actin was used as a loading control. **(c)** Immunostaining for SPINK1 in SPINK1-positive (WiDr, SK-MEL-173 and CAPAN-1) and SPINK1-negative (PC3) cell line, 22RV1 cells were used for no primary (1°) antibody control. Scale bar represents 10µm. **(d)** QPCR data showing relative expression of *SPINK1* and *KLK3* in 22RV1 cells stimulated with R1881 at various concentrations. **(e)** Same as in (d) except, VCaP cells were used. **(f)** QPCR data showing relative expression of androgen activated genes in R1881 (10 nM) stimulated 22RV1 cells. **(g)** Same as in (f), except VCaP cells were used. **(h)** QPCR data showing relative expression of androgen repressed genes in R1881 (10 nM) stimulated 22RV1 cells. **(i)** Same as in (h), except VCaP cells were used. **(j)** Quantification of SPINK1 levels in the cell lysate (CL) and conditioned media (CM) of 22RV1 cells treated with R1881 (10nM), determined using enzyme-linked immunosorbent assay (ELISA). **(k)** Same as in (j) except, VCaP cells treated with enzalutamide. **(l)** QPCR data showing relative expression of *SPINK1*, *KLK3* and *ERG* in bicalutamide treated VCaP cells. Immunoblot analysis showing SPINK1 expression in the same cells (top). β-actin was used as a loading control. **(m)** Immunostaining for SPINK1 and ERG using same cells as in (l). F-actin and nucleus was stained using TRITC-phalloidin and DAPI respectively. Scale bar represents 10µm. **(n)** Quantification of SPINK1 immunofluorescence images of same cells as in (m), represented as mean ± SD using ten fields per condition.
Experiments were performed with n=3 biologically independent samples; data represents mean±SEM. For panels (**d**, **e**, **j**, **k**, **l**) two-way ANOVA, Dunnett's multiple comparison test; (**f-i**) two-tailed unpaired Student's *t*-test; (**n**) one-way ANOVA, Tukey's multiple comparison test was applied. *$P \leq 0.05$ and **$P \leq 0.001$. Source data for (**b**, **l**) are provided as a Source Data file.

Supplementary Figure 3

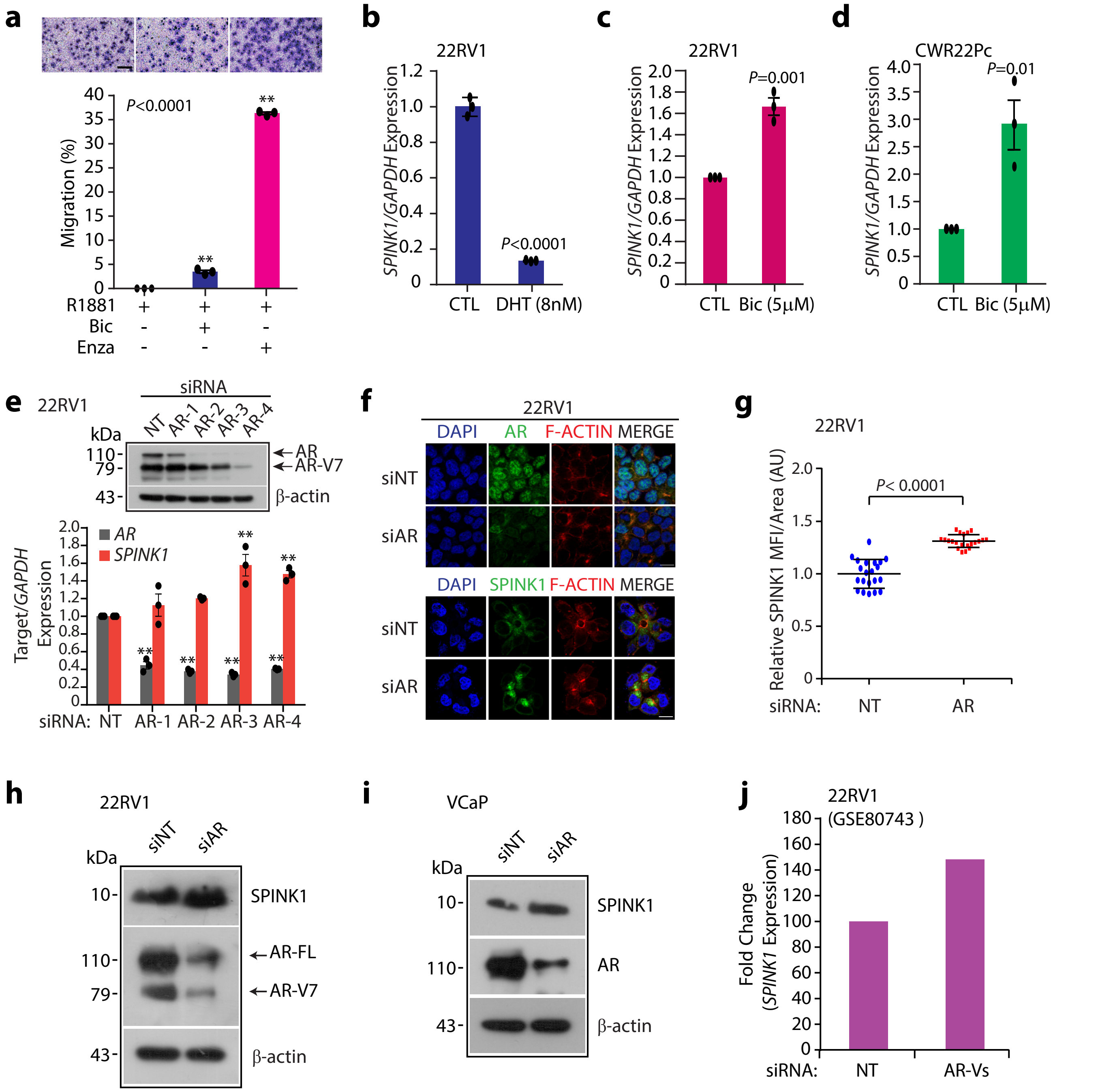

Supplementary Figure 3. Androgen signaling negatively regulates SPINK1 expression in PCa cells.
(a) Bar plot showing boyden chamber Matrigel invasion assay using bicalutamide (50μM) and enzalutamide (10μM) treated VCaP cells with or without androgen (R1881; 10nM) stimulation. Scale bar represents 200μm. **(b)** QPCR data showing relative expression of *SPINK1* in 22RV1 cells cultured in the presence of androgen (8 nM DHT) for 2 months. **(c)** Same as in (b), except 22RV1 cells were cultured in the presence of bicalutamide (5μM) for 2 months. **(d)** Same as in (c), except CWR22Pc cells were used. **(e)** QPCR data showing relative expression of *AR* and *SPINK1* in siRNA mediated *AR* silenced 22RV1 cells with respect to control cells (NT). Immunoblot analysis for AR levels using same cells (top). β-actin was used as a loading control. **(f)** Immunostaining for AR and SPINK1 in siRNA mediated *AR* silenced 22RV1 cells with respect to control. F-actin and nucleus was stained using TRITC-phalloidin and DAPI respectively. Scale bar represents 10μm. **(g)** Quantification of SPINK1 immunofluorescence images using same cells as in (f), data represented as mean±SD using ten fields per condition. **(h)** Immunoblot analysis for SPINK1 and AR level in siRNA mediated *AR* silenced 22RV1 cells with respect to control. β-actin was used as a loading control. **(i)** Same as in (h), except siRNA mediated *AR* silenced VCaP cells were used. **(j)** Bar graph showing fold change of *SPINK1* expression in 22RV1 cells with siRNA mediated knockdown of AR splice variants (*AR-V1*, *AR-V3*, *AR-V4* and *AR-V7*) as compared to the control (*NT*) (GSE80473).
Experiments were performed with n=3 biologically independent samples; data represents mean±SEM. For panel (**a**) one-way ANOVA, Dunnett's multiple comparison test; (**b-d**, **g**) two-tailed unpaired Student's *t*-test; (**e**) two-way ANOVA, Dunnett's multiple comparison test was applied. *$P\leq 0.05$ and **$P\leq 0.001$. For panel (**g**) scatter plots are represented as mean±SD. Source data for (**e**, **h**, **i**) are provided as a Source Data file.

Supplementary Figure 4

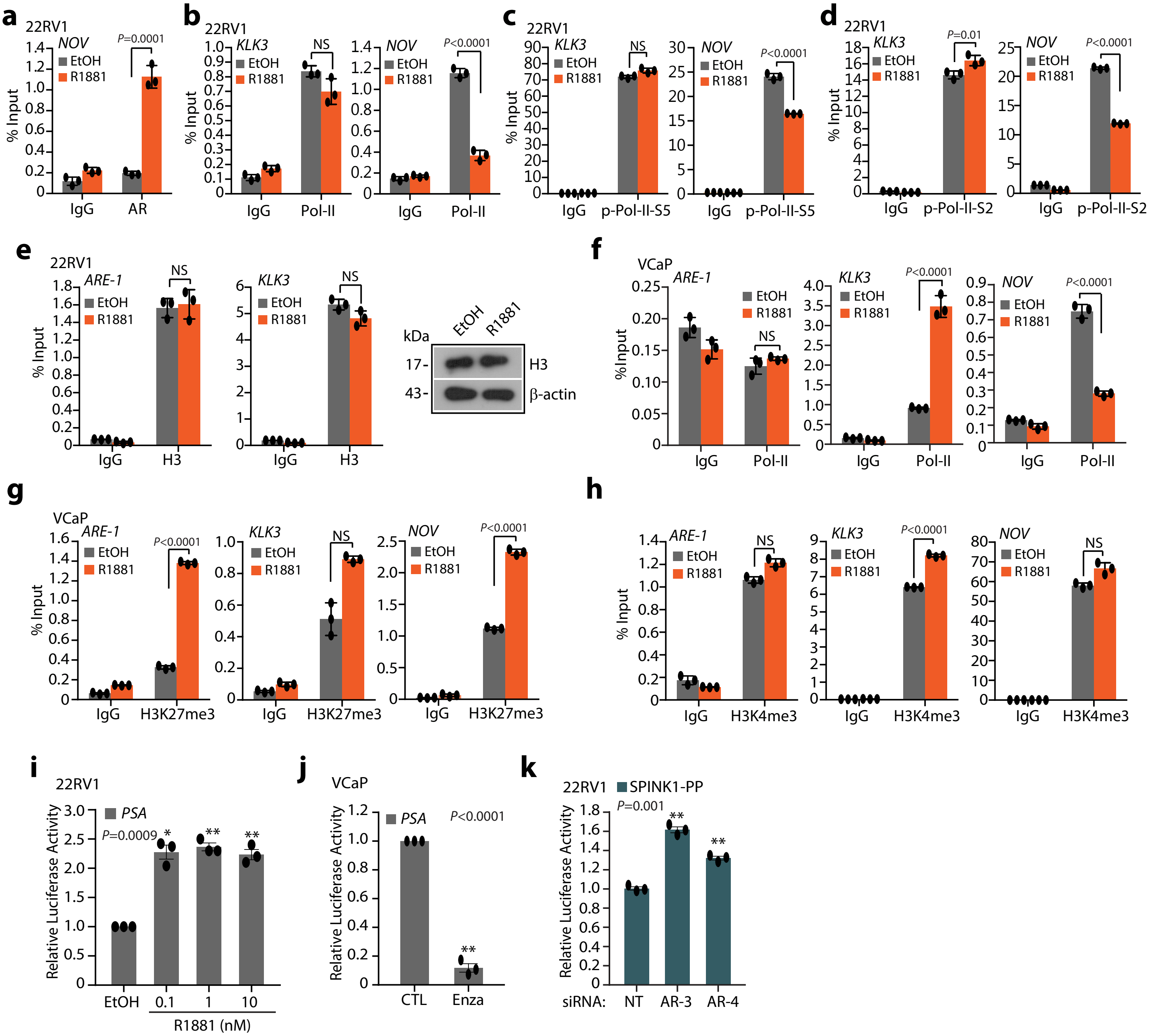

Supplementary Figure 4. AR is recruited on *SPINK1* promoter upon androgen stimulation.
(a) ChIP-qPCR data for AR occupancy on the *NOV* promoter in androgen (R1881; 10 nM) stimulated 22RV1 cells. **(b)** Same cells as in (a) except, ChIP-qPCR for RNA Pol II occupancy on the *KLK3* and *NOV* promoter. **(c)** Same cells as in (a) except, ChIP-qPCR for RNA Pol II Ser5 phosphorylation on the *KLK3* and *NOV* promoter. **(d)** Same cells as in (a) except, ChIP-qPCR for RNA Pol II Ser2 phosphorylation on the *KLK3* and *NOV* promoter. **(e)** Same cells as in (a) except, ChIP-qPCR for total Histone H3 (H3)on the *SPINK1* and *KLK3* promoter. Immunoblot analysis for H3 levels (right panel). β-actin was used as a loading control. **(f)** ChIP-qPCR data for RNA Pol II occupancy on the *SPINK1*, *KLK3* and *NOV* promoter in androgen (R1881; 10 nM) stimulated VCaP cells. **(g)** Same cells as in (f) except, ChIP-qPCR for presence of Histone 3 lysine 27 trimethylation (H3K27me3) marks on the *SPINK1, KLK3* and *NOV* promoters. **(h)** Same cells as in (f) except, ChIP-qPCR for Histone 3 lysine 4 trimethylation (H3K4me3) marks on the *SPINK1, KLK3* and *NOV* promoters. **(i)** Luciferase reporter activity of *PSA* promoter construct in 22RV1 cells treated with different concentrations of R1881. **(j)** Same as in (i) except, enzalutamide (10µM) treated VCaP cells. **(k)** Same as in (i) except, proximal promoter of *SPINK1* (SPINK1-PP) using siRNA mediated *AR* knockdown 22RV1 cells.
Experiments were performed with n=3 biologically independent samples; data represents mean ± SEM. For panels (**a-h, j**) two-tailed unpaired Student's *t*-test; (**i**, **k**) one-way ANOVA, Dunnett's multiple comparison test were applied. *$P\leq 0.05$ and **$P\leq 0.001$. Source data for (**e**) is provided as a Source Data file.

Supplementary Figure 5

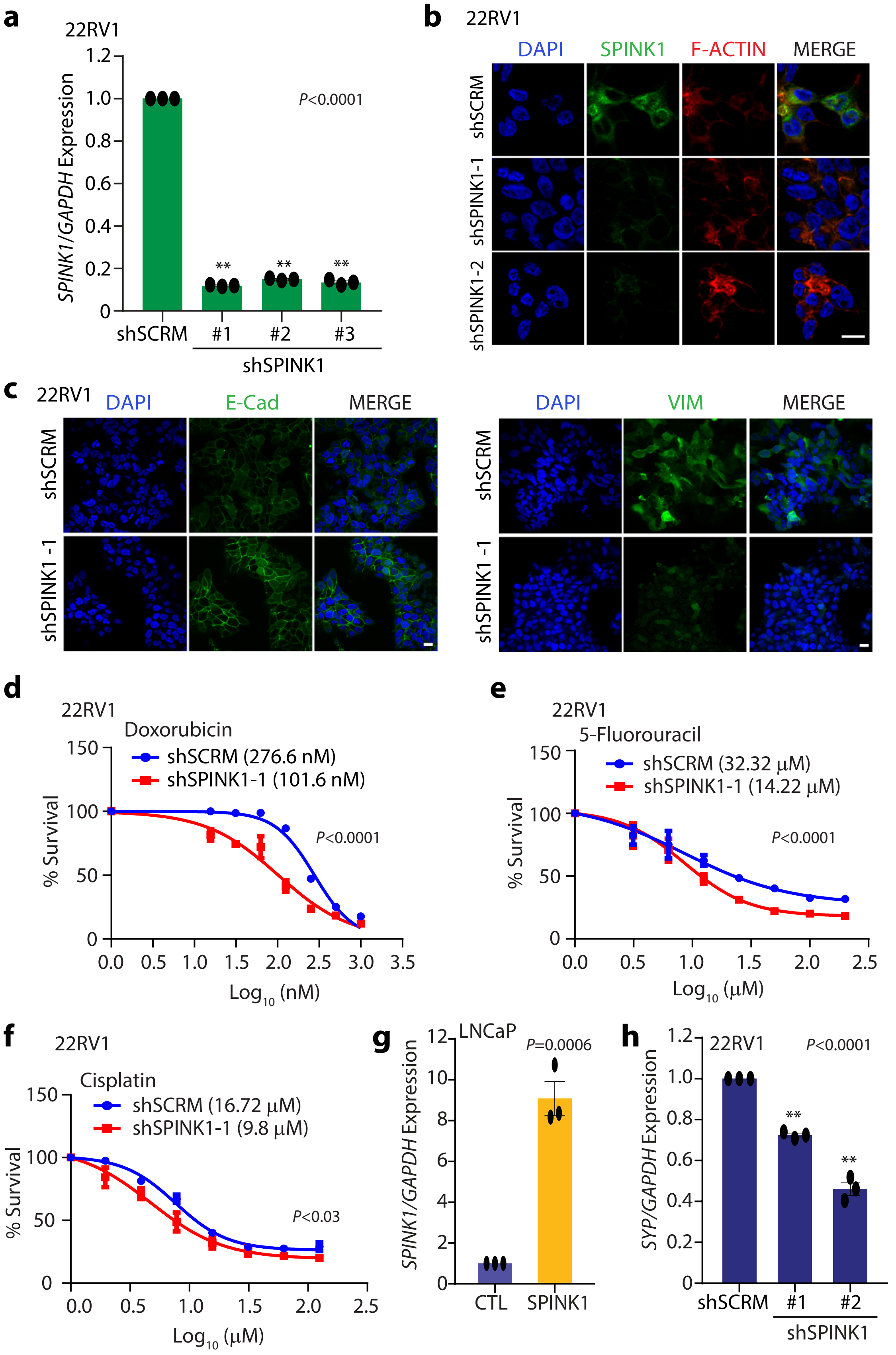

Supplementary Figure 5. Knockdown of *SPINK1* attenuates epithelial–mesenchymal transition and stemness in PCa cells. **(a)** QPCR data showing relative expression of *SPINK1* in stable *SPINK1*-silenced 22RV1 cells using three independent shRNA against *SPINK1*. **(b)** Immunostaining for SPINK1 using same cells as in (a). F-actin and nucleus was stained using TRITC-phalloidin and DAPI respectively. Scale bar represents 10µm. **(c)** Immunostaining for E-Cad (left) and VIM (right) using same cells as in (a). Scale bar represents 20µm **(d)** Dose-response curve with a range of doxorubicin concentration using control 22RV1-shSCRM (IC_{50}= 276.6 nM, 95% CI = 244.7 to 312.8 nM) and 22RV1-shSPINK1 (IC_{50}= 101.6 nM, 95% CI = 84.33 to 122.4 nM) cells. **(e)** Same as in (d) except, 5-fluoro-uracil using control 22RV1-shSCRM (IC_{50}= 32.32 µM, 95% CI = 24.51 to 42.63 µM) and 22RV1-shSPINK1 (IC_{50}= 14.22 µM, 95% CI = 11.14 to 18.15 µM) cells. **(f)** Same as in (d) except, cisplatin using control 22RV1-shSCRM (IC_{50}= 16.72 µM, 95% CI = 12.32 to 22.70 µM) and 22RV1-shSPINK1 (IC_{50}=9.8 µM, 95% CI = 7.491 to 12.84 µM) cells. **(g)** QPCR data showing relative expression of *SPINK1* in stable SPINK1 overexpressing LNCaP cells. **(h)** QPCR data showing relative expression of *SYP* in 22RV1-shSCRM and 22RV1-shSPINK1 cells. Experiments were performed with n=3 biologically independent samples; data represents mean ± SEM. For panels (**a**, **h**) one-way ANOVA, Dunnett's multiple comparison test; (**d-g**) two tailed unpaired students *t*-test. *$P \leq 0.05$ and **$P \leq 0.001$.

Supplementary Figure 6

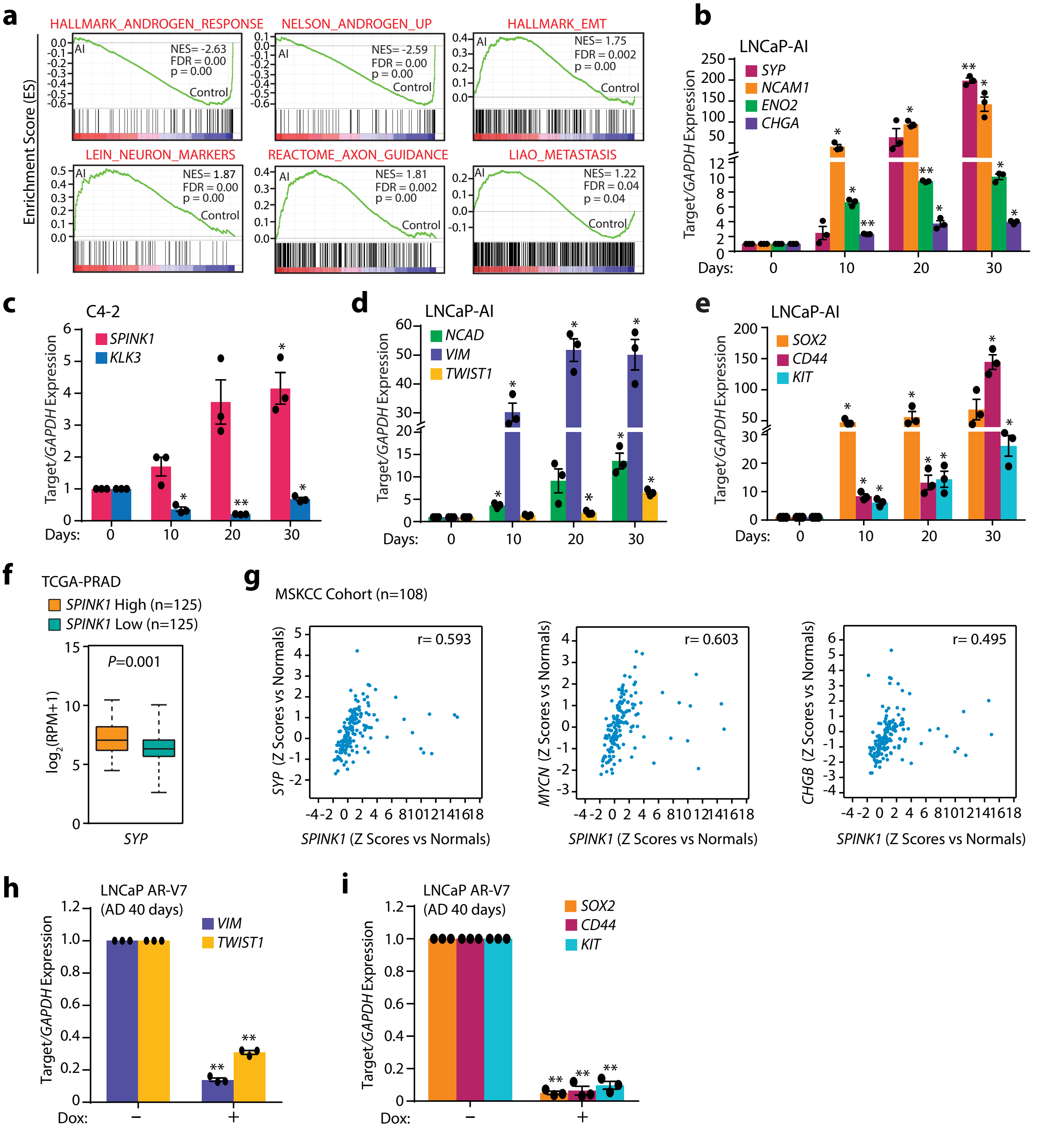

Supplementary Figure 6. SPINK1 expression is positively correlated with neuroendocrine prostate cancer (NEPC) signature genes.

(a) Gene Set Enrichment Analysis (GSEA) plots showing gene signatures associated with androgen signaling, neuroendocrine phenotype and EMT with the corresponding statistical metrics in long-term androgen deprived LNCaP cells relative to control cells (GSE8702). **(b)** QPCR data showing relative expression of *SYP*, *CHGA*, *ENO2*, and *NCAM1* in long-term androgen deprived (30 days) LNCaP-AI cells. **(c)** Same as in (b), except relative expression of *SPINK1* and *KLK3* in long-term androgen deprived C4-2 cells, a metastatic subline derived from LNCaP-C4. **(d)** Same as in (b), except relative expression of *NCAD*, *VIM*, and *TWIST*. **(e)** Same as in (b), except relative expression of *CD44*, *SOX2*, *KIT*. **(f)** Expression of *SYP* in *SPINK1* high (n=125) and *SPINK1* low (n=125) PCa patients, stratified by employing quartile-based normalization of *SPINK1* expression in TCGA-PRAD dataset. Transcripts level shown as $\log_2$ (RPM+1). **(g)** Correlation plots between *SPINK1* and *SYP*, *CHGB* and *MYCN* mRNA Z-score in MSKCC PCa cohort (n=108) analysed using cBioportal. Spearman's correlation coefficient (r) is indicated. **(h)** QPCR data showing relative expression of *VIM* and *TWIST* in long-term androgen deprived (AD; 40 days) doxycycline-inducible LNCaP AR-V7 overexpressing cells, cultured in presence of doxycycline for 40 day. **(i)** Same as in (h), except relative expression of *CD44*, *SOX2* and *KIT*.

Experiments were performed with n=3 biologically independent samples; data represents mean ± SEM. For panels (**b-f**) two-tailed unpaired Student's *t*-test; (**h-i**) two-way ANOVA, Sidak's multiple comparison test was applied. $^*P \leq 0.05$ and $^{**}P \leq 0.001$. For panel (**f**) data is is presented as box-and-whisker plots with median, where the box extends from 25th-75th percentile, and whiskers ranges from minimum and maximum values.

Supplementary Figure 7

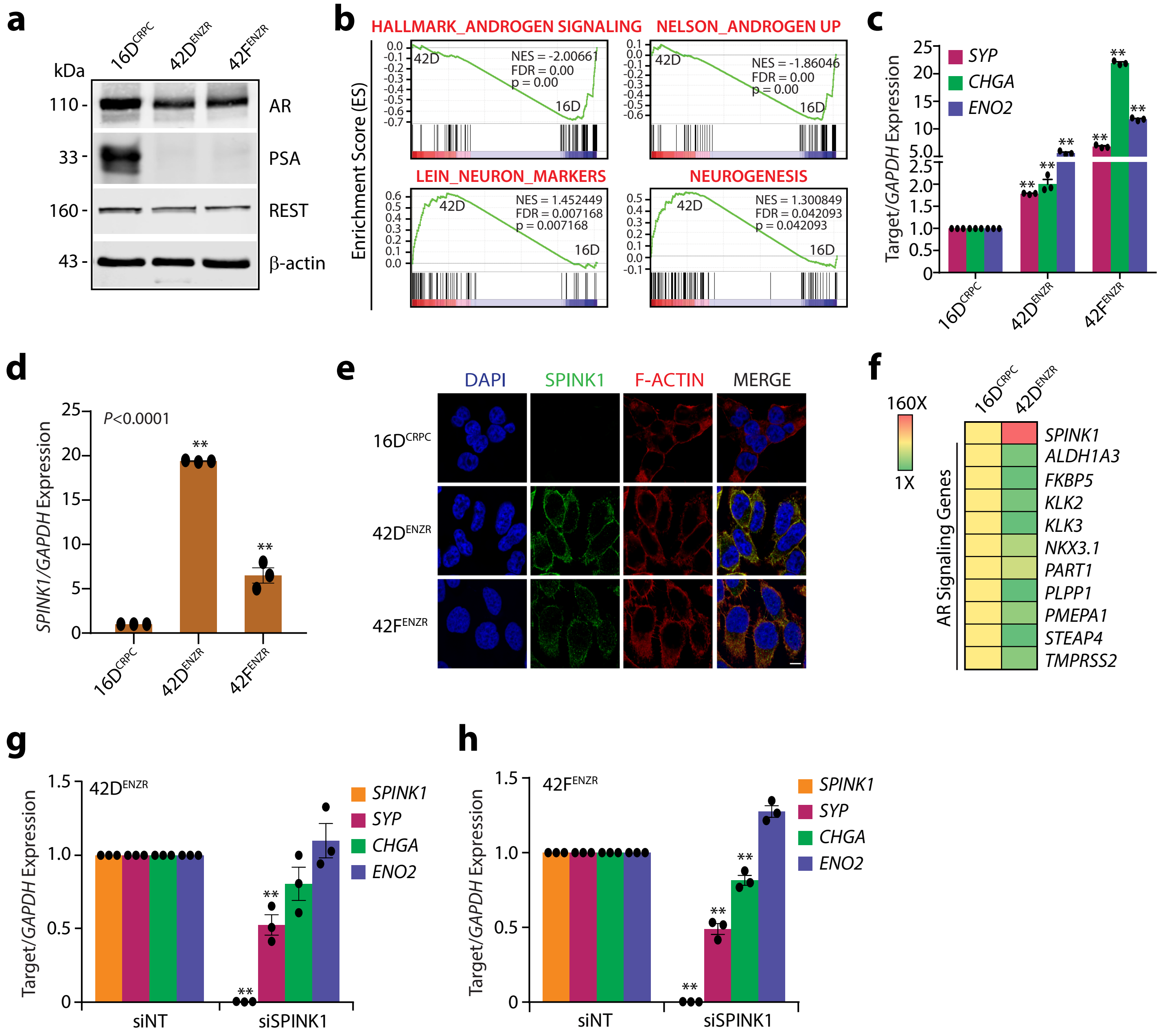

Supplementary Figure 7. Enzalutamide-resistant tumors show higher expression of SPINK1.
(a) Immunoblot assay for AR, PSA and REST levels in LNCaP xenografts derivatives, namely 16D^{CRPC} and enzalutamide resistant 42D^{ENZR} and 42F^{ENZR} cell lines. β-actin was used as a loading control. **(b)** Gene Set Enrichment Analysis (GSEA) plots showing gene signatures associated with androgen signaling and neuroendocrine phenotype with the corresponding statistical metrics in the 42D^{ENZR} cells compared to 16D^{CRPC}. **(c)** QPCR data showing relative expression of *SYP*, *CHGA* and *ENO2* in 16D^{CRPC}, 42D^{ENZR} and 42F^{ENZR} cells. **(d)** Same as in (c), except expression of *SPINK1*. **(e)** Immunostaining for SPINK1 using the same cells as in (c). Scale bar represents 10µm. **(f)** Heatmap representing fold increase in *SPINK1* transcript versus AR target genes in 42D^{ENZR} cells compared to 16D^{CRPC}. Data is plotted as reads per million. **(g)** QPCR data showing relative expression of *SPINK1*, *SYP*, *CHGA* and *ENO2* in siRNA mediated *SPINK1* silenced 42D^{ENZR} cells. **(h)** Same as in (g), except 42F^{ENZR} cells were used.
Experiments were performed with n=3 biologically independent samples; data represents mean ± SEM. For panel (**c**) two-way ANOVA, Dunnett's multiple comparison test; (**d**) one-way ANOVA, Dunnett's multiple comparison test; (**g-h**) two-way ANOVA, Sidak's multiple comparisons test was applied. *$P \leq 0.05$ and **$P \leq 0.001$. Source data for (**a**) are provided as a Source Data file.

Supplementary Figure 8

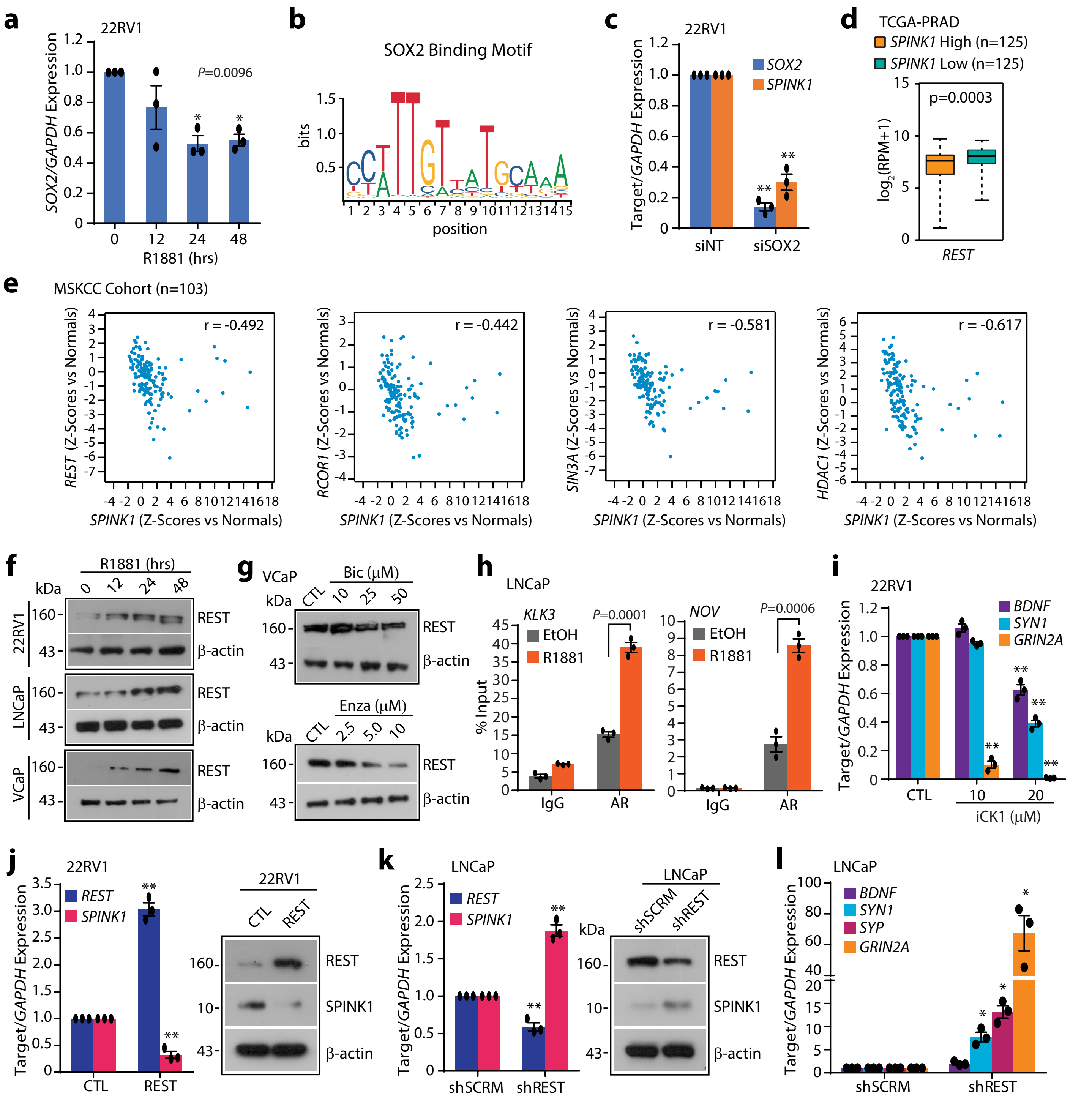

Supplementary Figure 8. REST serves as a transcriptional co-repressor of AR and negatively regulates *SPINK1* expression.
(a) QPCR data showing relative expression of *SOX2* in androgen (R1881; 10 nM) stimulated 22RV1 cells. **(b)** Schema showing SOX2 binding motif constructed from JASPAR database. **(c)** QPCR data showing relative expression of *SOX2 and SPINK1* in siRNA mediated *SOX2* silenced 22RV1 cells with respect to control. **(d)** Relative expression of *REST* across SPINK1 high (n=125) and SPINK1 low (n=125) PCa patients, stratified by employing quartile-based normalization of *SPINK1* expression in TCGA-PRAD dataset. Transcripts level shown as $\log_2$ (RPM+1). **(e)** Correlation plots between *SPINK1* and *REST*, *RCOR1*, *SIN3A* and *HDAC1* mRNA Z-Scores in MSKCC PCa cohort (n=103) analyzed using cBioPortal. Spearman's correlation coefficient (r) indicated. **(f)** Immunoblot analysis for REST level in 22RV1, LNCaP and VCaP cells upon androgen (R1881; 10 nM) stimulation. β-actin was used as a loading control. **(g)** Same as in (f), except anti-androgens (bicalutamide and enzalutamide) treated VCaP cells were used. β-actin was used as a loading control. **(h)** ChIP-qPCR data showing recruitment of AR on the *KLK3* and *NOV* promoters in androgen (R1881; 10 nM) stimulated LNCaP cells. **(i)** QPCR data showing relative expression of REST regulated genes, *BDNF*, *SYN1* and *GRIN2A* in 22RV1 cells treated with Casein Kinase 1 inhibitor (iCK1) at the indicated concentrations. **(j)** QPCR data showing relative expression of *REST* and *SPINK1* in stable REST overexpressing 22RV1 cells (left). Immunoblot assay for SPINK1 and REST using the same cells (right). β-actin was used as a loading control. **(k)** Same as in (j) except, stable *SPINK1*-silenced LNCaP cells were used. **(l)** QPCR data showing relative expression of REST regulated genes, *BDNF*, *SYN1*, *SYP* and *GRIN2A* using same cells as in (k).

Experiments were performed with n=3 biologically independent samples; data represents mean ± SEM. For panels (**a**) one-way ANOVA, Dunnett's multiple comparison test; (**c**) two-way ANOVA, Dunnett's multiple comparison test; (**d, h**) two-tailed unpaired Student's t-test; (**i-l**) two-way ANOVA, Sidak's multiple comparisons test was applied. **P*≤ 0.05 and ***P*≤ 0.001. For panel (**d**) data is presented as box-and-whisker plots with median, where the box extends from 25th-75th percentile, and whiskers ranges from minimum and maximum values. Source data for (**f**, **g**, **j**, **k**) are provided as a Source Data file.

Supplementary Figure 9

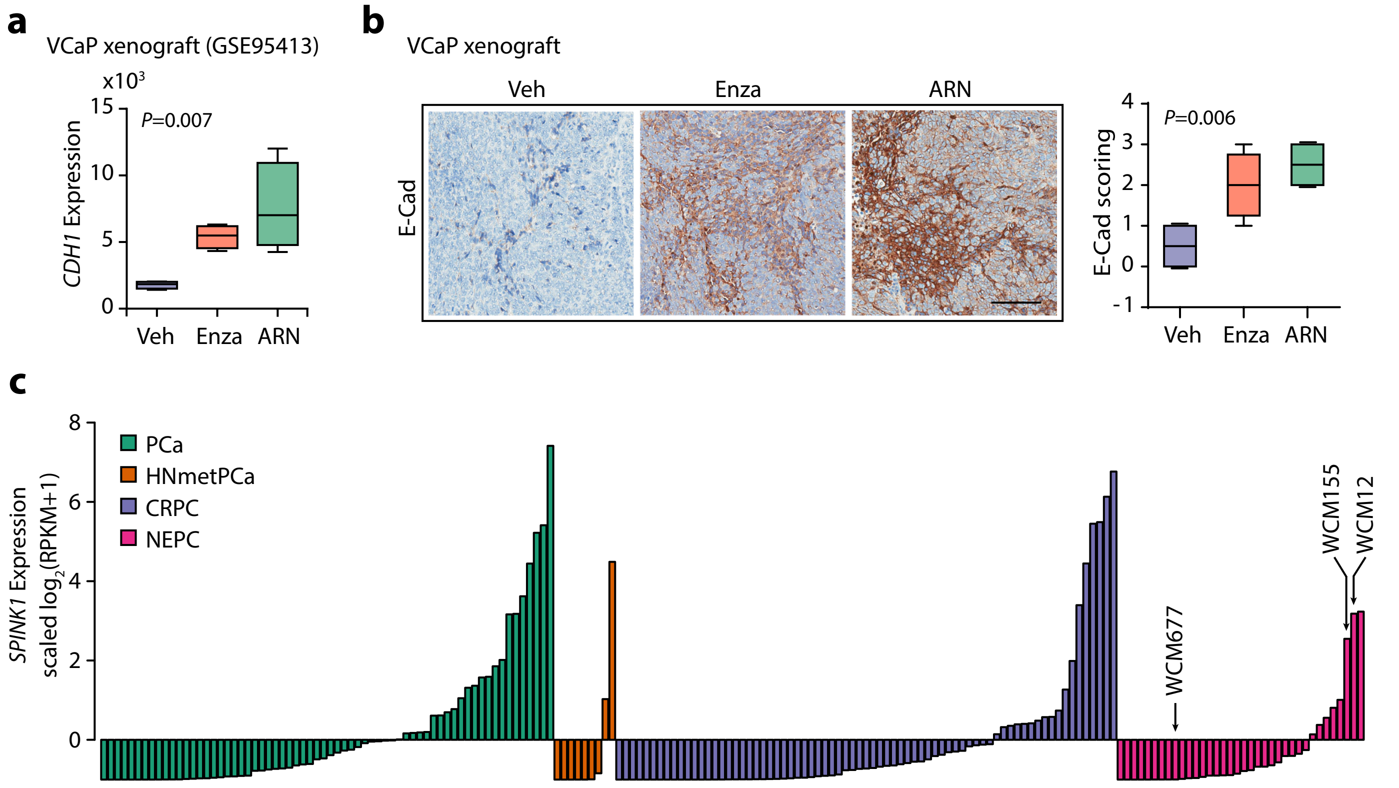

Supplementary Figure 9. SPINK1 expression in associated with NE-like features in ADT-administered mice and NEPC patients.
(a) Box plot depicting relative expression of *CDH1* transcripts (read counts) in VCaP tumors implanted orthotopically in orchiectomized mice, and administered with vehicle (n=4) or anti-androgens [enzalutamide (n=4) or ARN-509 (n=4)] treatment for 4 weeks (GSE95413). **(b)** Representative images of immunohistochemical staining for E-Cad (left panel) and quantification of the staining (right panel) in VCaP tumors treated with vehicle (n=4) or anti-androgens [enzalutamide (n=4) or ARN-509 (n=4)] for 4 weeks as in (a). Scale bar represents 100µm. **(c)** *SPINK1* expression in prostate adenocarcinoma (PCa, n=66), hormone naive metastatic prostate cancer (HNmetPCa, n=9), castrate-resistant prostate cancer (CRPC, n=73) and neuroendocrine prostate cancer (NEPC, n=36) clinical specimens in Beltran cohort.
Data are presented as box-and-whisker plots with median, where the box extends from 25th-75th percentile, and whiskers ranges from minimum and maximum values. For panels (**a**, **b**) one-way ANOVA, Dunnett's multiple comparisons test was applied.

Supplementary Table 1: Downregulated biological processes in 22RV1 shSPINK1 cells predicted by DAVID analysis.

Category	Term	Count	*P*- Value	Genes	Fold Enrichment	Bonferroni	Benjamini	FDR
GOTERM_BP_ DIRECT	GO:0007399~nervous system development	15	0.0016934	CXCL1, EFNB3, MAFB, MBD5, INHA, PCDHB12, MYT1, MDK, GJB1, PCDH18, SEMA6A, APBA2, APAF1,	2.651452151	0.95832451	0.958324511	2.85061
GOTERM_BP_ DIRECT	GO:0045944~positive regulation of transcription from RNA polymerase II promoter	33	0.0034502	E2F4, E2F5, HCFC1, ELK1, NFKB2, SKAP1, SRF, TGFB1, CALCOCO1, NR1H2, HOXA2, TEF, POU4F2, NRG1, ZNF564, EGFR, ARHGEF2, RARG, RXRB, MAFB, NODAL, NKX2-8, TEAD2, KLF15, TET1, USF1, JUNB, SIRT2, SREBF2, SMO, CAMK1, TFAP2D, FOXC1	1.706551364	0.99846657	0.960841005	5.72689
GOTERM_BP_ DIRECT	GO:0034097~response to cytokine	6	0.0034698	MCL1, SYNJ1, NFKB2, BCL2L1, SRF, JUNB	5.853590518	0.99852223	0.886096791	5.7586
GOTERM_BP_ DIRECT	GO:0048143~astrocyte activation	3	0.0037024	EGFR, SMO, ADORA2A	30.43867069	0.99904596	0.824251675	6.13315
GOTERM_BP_ DIRECT	GO:0048384~retinoic acid receptor signaling	4	0.0049298	NR1H2, RARG, ACTN4, RXRB	11.27358174	0.99990543	0.843271145	8.08702
GOTERM_BP_ DIRECT	GO:0010507~negative regulation of autophagy	5	0.0052552	MCL1, RNF5, BCL2L1, SIRT2, MT3	7.045988587	0.99994878	0.807291349	8.59856
GOTERM_BP_ DIRECT	GO:2001240~negative regulation of extrinsic apoptotic signaling	5	0.0058025	MCL1, STRADB, BCL2L1, CX3CL1, NRG1	6.855556463	0.99998175	0.789606802	9.45296
GOTERM_BP_ DIRECT	GO:0006357~regulation of transcription from RNA polymerase II	18	0.0065717	NFE2, BATF2, TBX10, NFKBID, MAFB, TADA1, NFKB2, ZBED8, TCEAL2, USF1, JUNB, FEV, MED30,	2.07065787	0.99999572	0.786758291	10.6409
GOTERM_BP_ DIRECT	GO:0001666~response to hypoxia	10	0.0070417	CYBA, ACTN4, RYR1, CYTB, CHRNA7, APAF1, USF1, SRF, TGFB1,	2.949483594	0.99999824	0.770580637	11.3595
GOTERM_BP_ DIRECT	GO:0042981~regulation of apoptotic process	11	0.009646	PEA15, MCL1, ACTN4, CASP8, APAF1, GAS1, INHA, BCL2L1,	2.619916883	0.99999999	0.837551712	15.244
GOTERM_BP_ DIRECT	GO:0048012~hepatocyte growth factor	3	0.0126525	HPN, MST1R, SIRT2	16.91037261	1	0.885871225	19.5291
GOTERM_BP_ DIRECT	GO:0032930~positive regulation of	3	0.0126525	EGFR, CYBA, TGFB1	16.91037261	1	0.885871225	19.5291
GOTERM_BP_ DIRECT	GO:0045930~negative regulation of mitotic	4	0.0139929	EGFR, FOXC1, GAS1, TGFB1	7.804787358	1	0.889400469	21.3731
GOTERM_BP_ DIRECT	GO:0019827~stem cell population	5	0.016594	MED30, NODAL, LIN28A, TET1, ZNF358	5.073111782	1	0.910493435	24.8384
GOTERM_BP_ DIRECT	GO:0030203~glycosaminoglycan metabolic	4	0.0188359	GPC5, CHST9, FOXC1, SDC3	6.997395562	1	0.921662288	27.7093
GOTERM_BP_ DIRECT	GO:0045773~positive regulation of axon	4	0.0206309	ANAPC2, NRG1, SRF, DISC1	6.764149043	1	0.926158438	29.9331
GOTERM_BP_ DIRECT	GO:0045766~positive regulation of	7	0.0260231	CCL11, CD34, NODAL, CXCR2, CHRNA7, CX3CL1, ANXA3	3.087981085	1	0.954496499	36.2326
GOTERM_BP_	GO:0043588~skin	4	0.0287174	JUP, RYR1, ITGA3, SLITRK5	5.968366803	1	0.959793655	39.1767
GOTERM_BP_ DIRECT	GO:0010628~positive regulation of gene	11	0.0347939	SMO, HPN, CD34, PRKAG1, HCFC1, ITGA3, WNT6, TGFB1, CALCOCO1,	2.129932428	1	0.975000956	45.3535
GOTERM_BP_	GO:0038128~ERBB2	4	0.0382437	EGFR, NRG4, PTPRR, NRG1	5.340117666	1	0.978680006	48.5923
GOTERM_BP_ DIRECT	GO:0006695~cholesterol biosynthetic process	4	0.0382437	TM7SF2, MSMO1, LSS, IDI1	5.340117666	1	0.978680006	48.5923
GOTERM_BP_ DIRECT	GO:0042307~positive regulation of protein	3	0.0385348	JUP, SMO, TGFB1	9.512084592	1	0.97487987	48.8572
GOTERM_BP_ DIRECT	GO:0048168~regulation of neuronal synaptic	3	0.0385348	SYP, ARC, DBN1	9.512084592	1	0.97487987	48.8572
GOTERM_BP_ DIRECT	GO:0007010~cytoskeleton organization	8	0.0400267	CCL11, DMTN, SEMA6A, FMNL2, ARC, MAEA, CFL1, PSTPIP2	2.520800886	1	0.973938627	50.1945
GOTERM_BP_ DIRECT	GO:0050908~detection of light stimulus	3	0.043117	SEMA5B, GNAT2, CACNA2D4	8.952550204	1	0.976630451	52.8608
GOTERM_BP_ DIRECT	GO:0007268~chemical synaptic transmission	10	0.0481264	SSTR5, PDE7B, NPY, SSTR1, HTR6, SDCBP, MINK1, APBA2, SLITRK5,	2.113796576	1	0.982063137	56.8992
GOTERM_BP_ DIRECT	GO:0071300~cellular response to retinoic	5	0.0488791	HOXA2, RARG, TEAD2, SNRNP70, WNT6	3.623651273	1	0.980062683	57.4771

Supplementary Table 2: List of Primers.

Quantitative PCR (qPCR) Primers			
**S. No.**	**Gene Name**	**Primer Name**	**Sequence (5'-3')**
1	*SPINK1*	qSPINK1_FP	GTCTGTGGGACTGATGGAAATA
		qSPINK1_RP	GAGGATAGAAGTCTGGCGTTTC
2	*AR*	qAR_FP	AATCCCACATCCTGCTCAAG
		qAR_RP	GAGTCCAGGAGCTTGGTGAG
3	*KLK3*	qPSA_FP	GTCTGCGGCGGTGTTCTG
		qPSA_RP	TGCCGACCCAGCAAGATC
4	*TMPRSS2*	qTMPRSS2_FP	CAGGAGTGTACGGGAATGTGATGGT
		qTMPRSS2_RP	GATTAGCCGTCTGCCCTCATTTGT
5	*ERG*	qERG_FP	CGCAGAGTTATCGTGCCAGCAGAT
		qERG_RP	CCATATTCTTTCACCGCCCACTCC
6	*FKBP5*	qFKBP_FP	GCAGGCGGTGATTCAGTAT
		qFKBP_RP	AGGTTCAGAAAGGCAGCAA
7	*SOX2*	qSOX2_FP	CATGGGTTCGGTGGTCAAG
		qSOX2_RP	TGATCATGTCCCGGAGGT
8	*SYP*	qSYP_FP	CCTCTGCTATGTCTGTGATGTC
		qSYP_RP	TACCGAAGGGTTTGGGAAAG
9	*CHGA*	qCHGA_FP	CTGAACACAGGCAGCTTTCTA
		qCHGA_RP	CAGTCAGGAGTTCTCAGCTTTC
10	*ENO2*	qENO2_FP	AGTTCACAGCCAATGTAGGG
		qENO2_RP	CCGATCTGGTTGACCTTGAG
11	*NCAM1*	qNCAM1_FP	ACCAGACTGCCATGGAATTAG
		qNCAM1_RP	GCTGATGTTCCGGGTAGAAG
12	*CDH2*	qN-CAD_FP	TGACTCCCTGTTAGTGTTTGAC
		qN-CAD_RP	CCCAGTCGTTCAGGTAATCATAG
13	*VIMENTIN*	qVIM_FP	GATTCACTCCCTCTGGTTGATAC
		qVIM_RP	GTCATCGTGATGCTGAGAAGT
14	*TWIST1*	qTWIST1_FP	CCAGGTACATCGACTTCCTCTA
		qTWIST1_RP	CCATCCTCCAGACCGAGAA
15	*CD44*	qCD44_FP	CAGCACTTCAGGAGGTTACAT
		qCD44_RP	GTAGCAGGGATTCTGTCTGTG
16	*KIT*	qKIT_FP	CAAGGCTTCTCCAATTCTGC
		qKIT_RP	TGCAGTGGTCCACAGAAGAG
17	*REST*	qREST_FP	AGAGAAGAGGCATCAGGAGA
		qREST_RP	ATTAGCAGCAAGACCAGGTAG
18	*BDNF*	qBDNF_FP	GCGTGTGTGACAGTATTAGT
		qBDNF_RP	CTGGGTAGTTCGGCACTGGG
19	*SYN1*	qSYN1_FP	GCACGTCCTGGCTGGGTTTCTGGG
		qSYN1_RP	AGGCTACCCGTCAGACATCCGTCTC
20	*GRIN2A*	qGRIN2A-FP	GTGGTCTATCAACGGGCAGT
		qGRIN2A-RP	AGGTGAGACGGTGCCATTAC
21	*GAPDH*	qGAPDH_FP	TGCACCACCAACTGCTTAGC
		qGAPDH_RP	GGCATGGACTGTGGTCATGAG

22	*KLK2*	qKLK2_FP	CTCCATCTCCTGTCCAATGAC
		qKLK2_RP	AAGTGTCTTTACCACCTGTCC
23	*ACSL3*	qACSL3_FP	GGGCCAAAGTGTGACAATGG
		qACSL3_RP	CGGGTTCAAACTCTCCAATATCC
24	*PIAS1*	qPIAS1_FP	AGTCTTCCACATCAAGCATCTC
		qPIAS1_RP	GGATGCCTATAGTCTTGGATGAG
25	*PPAP2A*	qPPAP2A_FP	CCACACTGCAATTTGGTCTTG
		qPPAP2A_RP	CCCTGAATGAGTCCAGTCAAC
26	*SLC26A2*	qSLC26A2_FP	GGAAGAAGGCAGCAAAGAGA
		qSLC26A2_RP	CTCCAAGGGATCATGGGAAAG
27	*STK39*	qSTK39_FP	GGTCAAGTGGTCACCTTCATAA
		qSTK39_RP	CTTTCCCTTCTTCGCTCTTCTC
28	*STEAP4*	qSTEAP4_FP	GCCTGGCTCAGTGATTCATA
		qSTEAP4_RP	GCTAACAGATGGCAAAGAAGTG
29	*DDC*	qDDC_FP	GCCCCTACTTCTTCGCCTAC
		qDDC_RP	CACAGTCTCCAGCTCTGTGC
30	*OPRK1*	qOPRK1_FP	GCTGGACTCCCATTCACATATT
		qOPRK1_RP	GGATTCAGGCTACTGTTGGTATAG
31	*NOV*	qNOV_FP	ACCGTCAATGTGAGATGCTG
		qNOV_RP	TCTTGAACTGCAGGTGGATG
32	*SERPINI1*	qSERPINI1_FP	CTTGCTACTCTGGAGCCATTAG
		qSERPINI1_RP	ACCTGGGCAGGTATACTTCTA

**Chromatin Immunoprecipitation-Quantitative PCR (ChIP-qPCR) Primers**			
**S. No.**	**Gene Name**	**Primer Name**	**Sequence (5'-3')**
1	*SPINK1*	ARE1_FP	GAGTCTATCTGGTAAGTGTTGCATA
		ARE1_RP	TCTCTCGAAGACTAGACTACATCAA
2	*SPINK1*	ARE2_FP	CTGCACTAGATTTAGACCCTCAC
		ARE2_RP	GAGAAACATCCAGGAGGACAC
3	*SPINK1*	ARE3_FP	GGCTGGCACCTGTTGAT
		ARE3_RP	CCAAATGTCAAGGAGGTCTCTAA
4	*SPINK1*	R1_FP	CCATCTGCCATATGACCCTTC
		R1_RP	AGATGCCTGTTACCTTCATGG
5	*SPINK1*	R2_FP	GGATTTCTCTGGTCAATGCAC
		R2_RP	CCTCACATGTCCAGGAATTA
6	*SPINK1*	R3_FP	AGGTGCCAGCCCAATATC
		R3_RP	GCATGTCTATCTTTGGGAACTC
7	*SPINK1*	S1_FP	GAGTCTATCTGGTAAGTGTTGCATA
		S1_RP	TCTCTCGAAGACTAGACTACATCAA
8	*SPINK1*	S2_FP	ACCCAGCACATATTCTCAGATG
		S2_RP	CTCAGTTCCCTAAAGCGTGTAT
9	*SPINK1*	S3_FP	ACATGGTCTTTGCCCTAGAAG
		S3_RP	AATACGTAATGTCTCCCTCTGC
10	*KLK3*	KLK3_FP	CCTAGATGAAGTCTCCATGAGCTACA
		KLK3_RP	GGGAGGGAGAGCTAGCACTTG

11	*NOV*	NOV_FP	GCTGAGTGGTTTCTCCTTGTC
		NOV_RP	ACACCAGACAGCATGAGCAG

**Mutagenesis Primers**			
**S. No.**	**Gene Name**	**Primer Name**	**Sequence (5'-3')**
1	*SPINK1*	MT1_FP	GCTCAAGCACGAGTGTCCTCCT
		MT1_RP	AGGAGGACACTCGTGCTTGAGC
2	*SPINK1*	MT2_FP	GCTCAAGCACGAGTGGCCTCCT
		MT2_RP	AGGAGGCCACTCGTGCTTGAGC
